# What do the patients with medication overuse headache expect from treatment and what are the preferred sources of information?

**DOI:** 10.1007/s10194-011-0301-0

**Published:** 2011-02-24

**Authors:** Michael Bjørn Russell

**Affiliations:** 1 Head and Neck Research Group, Research Centre, Akershus University Hospital, 1478 Lørenskog, Oslo, Norway; 2 Institute of Clinical Medicine, University of Oslo, Nordbyhagen, Oslo, Norway

Medication overuse headache (MOH) is a secondary chronic headache with a prevalence of 1.7% in the general population [1, 2]. The International Classification of Headache Disorders II (ICHD II) defines the overuse to be ≥15 days/month for simple analgesic, or ≥10 days/month for triptans, ergots, opioids, combination of analgesics or acute medications for >3 months [3, 4]. The socio-economic impact of MOH is enormous. Although direct data are missing it is probably one of the most costly illnesses, and the most costly headache. The current treatment strategy for MOH is detoxification. Detoxification is usually a time consuming and complex course usually requiring close interact between patient and physician, and relapse is frequent [5].

The pilot study by the Comoestas Consortium explore patients’ expectations and preferences for the treatment of MOH [6]. They hypothesize that compliance and satisfaction with treatment increase, if the patients have realistic expectations. This is an important study, since it explores the patients’ viewpoint rather than usual clinical trial primary and secondary end point such as reduction in headache days or efficacy of acute medication at 2 h.

The study included 65 consecutive patients with MOH referred to either of three tertiary headache centers. The participants filled in a questionnaire with three sections exploring their needs for headache information, preference for headache information and expectations of the headache treatment.

Personal contact either by direct or by telephone was both the preferred need and preference for headache information prior to e-mail, website information, and leaflets. This is in line with the fact that the patients did not benefit sufficiently from self-treatment. The internet provide enormous quantities of information of varied quality, which is difficult to analyze and use, while a leaflet might not cover the specific disease in quest. The preference for personal contact exist even thought the patients did not benefit sufficiently from the first and second-line treatment. Thus, the patients have more confidence in advice from health professionals than unsorted information from various other sources. The preference for person contact direct or by telephone prior to e-mail correspondance probably reflect a combination of speed and possibility for direct dialog.

The most common treatment expectations were reduction of the headache frequency and intensity. Fifteen and eighteen percent of the patients did not had those expectations, but since they showed up at the tertiary headache clinic, they probably had some other expectations such as a fast treatment or an effective previtive medication or it may be educational information. Overall 59% had expectation of a cure for their headache. This parameter showed the largest variation between the three centres, as 80% of the Italians, 45% of Germans and 50% Danes had this expectation. The difference was not significant probably to a type 2 error.

Finally this is an interesting and compelling pilot study that focus on the patients’ perspective. Hopefully the Comoestas Consortium will proceed on investigation the patients need and expectations in a larger populations in the future.
